# Leveraging insect-specific viruses to elucidate mosquito population structure and dynamics

**DOI:** 10.1371/journal.ppat.1011588

**Published:** 2023-08-31

**Authors:** Brandon D. Hollingsworth, Nathan D. Grubaugh, Brian P. Lazzaro, Courtney C. Murdock

**Affiliations:** 1 Department of Entomology, Cornell University, Ithaca, New York, United States of America; 2 Cornell Institute for Host Microbe Interaction and Disease, Cornell University, Ithaca, New York, United States of America; 3 Yale School of Public Health, New Haven, Connecticut, United States of America; 4 Yale University, New Haven, Connecticut, United States of America; 5 Northeast Regional Center for Excellence in Vector-borne Diseases, Cornell University, Ithaca, New York, United States of America; University of North Carolina at Chapel Hill School of Medicine, UNITED STATES

## Abstract

Several aspects of mosquito ecology that are important for vectored disease transmission and control have been difficult to measure at epidemiologically important scales in the field. In particular, the ability to describe mosquito population structure and movement rates has been hindered by difficulty in quantifying fine-scale genetic variation among populations. The mosquito virome represents a possible avenue for quantifying population structure and movement rates across multiple spatial scales. Mosquito viromes contain a diversity of viruses, including several insect-specific viruses (ISVs) and “core” viruses that have high prevalence across populations. To date, virome studies have focused on viral discovery and have only recently begun examining viral ecology. While nonpathogenic ISVs may be of little public health relevance themselves, they provide a possible route for quantifying mosquito population structure and dynamics. For example, vertically transmitted viruses could behave as a rapidly evolving extension of the host’s genome. It should be possible to apply established analytical methods to appropriate viral phylogenies and incidence data to generate novel approaches for estimating mosquito population structure and dispersal over epidemiologically relevant timescales. By studying the virome through the lens of spatial and genomic epidemiology, it may be possible to investigate otherwise cryptic aspects of mosquito ecology. A better understanding of mosquito population structure and dynamics are key for understanding mosquito-borne disease ecology and methods based on ISVs could provide a powerful tool for informing mosquito control programs.

## Introduction

Our understanding of mosquito-borne disease transmission and control has been drastically limited by gaps in knowledge around the vector’s behavior and ecology. These gaps cover critical areas such as the scale of mosquito dispersal, drivers of mosquito population structure, and genetic variation between proximate populations. These aspects of mosquito ecology impact not only our ability to predict the risk and dynamics of mosquito-borne disease but also the success of vector control programs. Mosquito dispersal affects the degree of connectivity and population structure that exists among mosquito populations in an area, with isolated populations becoming genetically differentiated due to local adaptation or drift events [1]. This genetic variation among populations can impact mosquito life history traits, vector competence, and host-feeding preferences [2,3], resulting in varying vectoral capacity between populations [4] and regional transmission dynamics [5]. Additionally, genetic variation among mosquito populations can have significant implications for vector-borne disease control, by impacting the size of disease clusters [6–8], the radius of insecticide treatments needed to prevent transmission [9,10], and the likelihood of reintroduction following local elimination of vector species [11–13]. Population structure has also been suggested as a major factor in the success of vector control using self-spreading elements (e.g., *Wolbachia* or gene drives) and will have implications for the coverage and numbers of mosquitoes that need to be released [14–16].

The appropriate spatial scale at which to quantify mosquito population structure and dispersal depends on the scale and relative importance of processes driving species dispersal. These processes can be divided into active and passive dispersal mechanisms. For species such as *Anopheles gambiae*, which can travel >500 m/day [17–19] without human assistance, these processes may operate on similar scales and result in comparable population structure. On the other hand, for species such as *Aedes aegypti*, which typically disperses <200 m in its life [20–24], the relative impacts of active and passive dispersal are expected to have major implications for population structure. In addition, having multiple scales of dispersal is expected to lead to multiple levels of population structure, with regional population structure possibly driven by passive dispersal and local dispersal within regions determined by active dispersal. However, the scale at which regional and local population structure occurs is species and environment dependent, and the relevant level at which to describe population structure is dependent on the questions being considered. While determining regional population structure over relatively long time periods is generally sufficient for studying longer-term evolutionary processes, epidemiological processes occur much more quickly and on a local scale, thus requiring quantification of local population structure.

**Fig 1:**
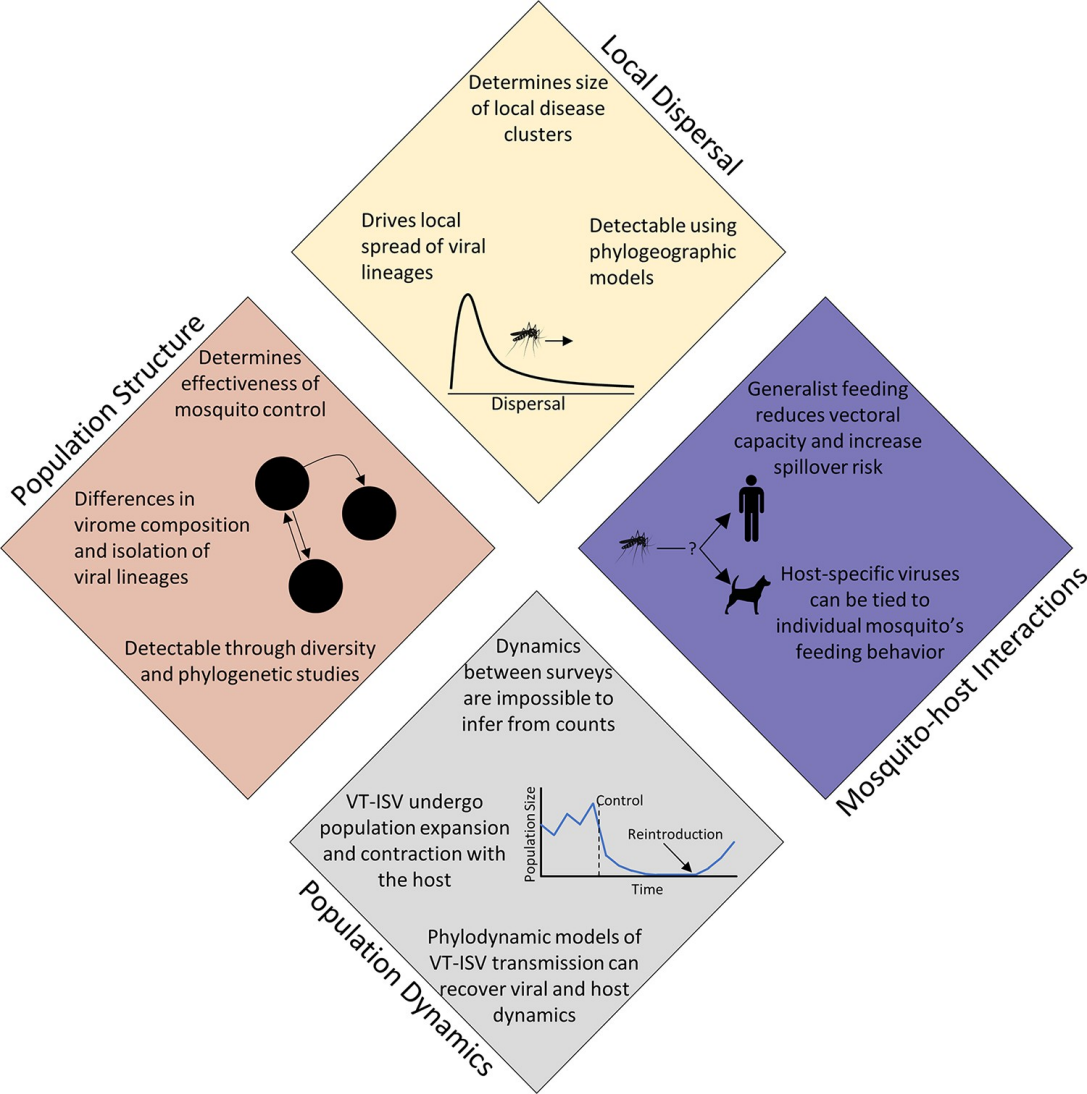
The mosquito virome is impacted by several aspects of the host’s ecology. Each of these aspects is expected to result in measurable changes in virome composition or phylogenies of individual viruses. This allows for inference of the host’s ecology from characterization of the host virome.

One of the fastest growing areas of research among vector species has been the study of viromes [25–31], the diverse collection of viruses found within a host species. This increased interest has partially been driven by the increased availability of metagenomic next-generation sequencing (mNGS) that has fundamentally shifted our understanding of the virosphere [25], the collection of viruses found worldwide. This work has shown a vast diversity of previously undiscovered viruses beyond pathogenic viruses of medical and veterinary importance and has emphasized the lack of clarity on the forces shaping viral ecology and evolution [25,26]. Concurrently, the number of published studies identifying nonpathogenic viruses in mosquitoes has increased exponentially from 2 papers published before 2014 to more than 172 papers published by 2023 [25,28,31], spanning at least 128 sampled species across 14 genera of mosquitoes [28,31–55]. In *Ae*. *aegypti* alone, there have been over 380 viruses identified, the vast majority of which are insect-specific viruses (ISVs) with no known pathogenicity [31]. Collectively, this represents the fourth largest collection of virome studies, eclipsed only by studies of humans, bats, and rodents [25]. These studies have focused almost exclusively on identifying novel viruses, with an interest in identifying those that either (1) are potential human pathogens or (2) could serve as a biocontrol through either antagonistic interactions with mosquito-borne pathogens (i.e., population replacement approaches) or pathogenic effects on the mosquito host (i.e., population suppression approaches) [27]. These studies have uncovered a diverse viral community in mosquitoes that is almost entirely unexplored [25,26,28–30]. Recently, studies have begun quantifying variation in the virome among species [35,53,56–58], mosquito sex [57], sampling locations [35,50,56], and local environment [56], showing that virome composition is the result of a complex interaction between mosquito species and their environment. While little is currently known about the biology and pathogenicity of newly discovered viruses, we can assume that most have at least a partial ability to escape the mosquito immune response, infect mosquito cells, replicate, and, if the infection is not a dead end, spread to new hosts. Because of this, these viruses can be expected to undergo dynamics like those of well-described viruses with the same basic transmission pathways. While the transmission dynamics of ISVs may be of little immediate relevance to public health, recent advances in spatial and genomic epidemiology (e.g., phylogeography [59]) make it possible to leverage ISVs to better understand mosquito population dynamics and structure. While medically relevant arboviruses (e.g., dengue virus) may not be well suited for discerning mosquito dynamics in this way, due to low infection rates among mosquitoes and complications due to multispecies transmission, ISVs could help elucidate several aspects of mosquito ecology (**Fig 1**).

Our goal here is to outline how the incidence and population genetics of ISVs can provide insight into key eco-evolutionary processes for mosquito-borne disease transmission across multiple scales. First, we will briefly provide an updated review on the current state of research on mosquito ISVs, tying genetic variation of these viruses to various eco-evolutionary processes that act on the host. Each of these processes has implications for mosquito-borne disease transmission and control but has historically been difficult to measure with more traditional approaches. In addition, we will discuss how statistical techniques commonly used in epidemiology can be adapted to leverage ISVs to improve our understanding of how environmental and seasonal heterogeneity affects mosquito population dynamics, mosquito-borne disease transmission, and the optimization of vector control.

## The diversity of viruses infecting mosquitoes

The availability of metagenomic viral screens have resulted in an explosion in the number of viral species that have been described in association with mosquitoes [25,26,28,30,31]. These viruses are believed to have been acquired from several sources, including blood meals, nectar feeding, and the larval habitat, with the majority believed to be ISVs (see [26–28,31] for more thorough review). One major result of the work on mosquito virome diversity is the concept of a “core” virome, a subset of a species’ virome composed of viruses that are found in the vast majority of individuals within a species [27,28,38,53,60,61]. Many of these viruses, such as *Aedes* Phasi Charoen-like virus (PCLV), have been identified in individuals from multiple continents and long-standing lab colonies (e.g., the Rockefeller line) and are believed to be maternally inherited ISVs with little or no fitness cost to the host [60,61]. Table 1 lists widespread ISVs, their host species, and distribution.

**Table 1:** Widely distributed mosquito ISVs. Table is based on [31] and contains all ISVs that have been reported on more than 4 continents.

Virus	Family	Hosts	Continents	Ref
Phasi Charoen-like virus	Phenuiviridae	*Aedes aegypti*, *Ae*. *albopictus*, *Anopheles* spp., *Culex quinquefasciatus*, *Haemagogus janthinomys*	Africa, Asia, Australia, North America, South America	[32,37,38,41,55,60,152–158]
Alphamesonivirus 1	Mesoniviridae	*Ae*. *aegypti*, *Ae*. *alboannulatus*, *Ae*. *albopictus*, *Ae*. *camptorhynchus*, *Ae*. *caspius*, *Ae*. *cinereus*, *Ae*. *dorsalis*, *Ae*. *hesperonotius*, *Ae*. *flavescens*, *Ae*. *flavidorsalis*, *Ae*. *notoscriptus*, *Ae*. *taeniorhynchus*, *Ae*. *treplicates*, *Ae*. *triseriatus*, *Ae*. *turneri*, *Ae*. *vexans*, *Ae*. *vigilax*, *Ae*. *spp*., *An*. *annulipes*, *An*. *sinensis*, *Armigeres subalbatus*, *Ar*. *obturbans*, *Coquillettidia xanthogaster*, *Cx*. *annulirostris*, *Cx*. *australicus*, *Cx*. *globocoxitus*, *Cx*. *modestus*, *Cx*. *pipiens*, *Cx*. *pullus*, *Cx*. *sitiens*, *Cx*. *tarsalis*, *Cx*. *torrentium*, *Cx*. *tritaeniorhynchus*, *Cx*. *vishnui*, *Cx*. spp.	Asia, Australia, Europe, North America, South America	[33,34,37,40,42,43,155,159–165]
Anopheles totivirus	Totiviridae	*Ae*. *aegypti*, *An*. *gambiae*	Africa, Asia, Australia, North America, South America	[32,37,51,166]
Cell fusing agent virus	Flaviviridae	*Ae*. *aegypti*, *Ae*. *caballus*, *Ae*. spp., *Cx*. spp.	Africa, Asia, Australia, North America, South America	[32,38,138,167–175]
*Culex pipiens*-associated tunisia virus	Unknown	*Ae*. *aegypti*, *Ae*. spp., *Ae*. *albopictus*, *Cx*. *erythrothorax*, *Cx*. *pipiens*, *Cx*. *tritaeniorhynchus*, *Cx*. *quinquefasciatus*	Africa, Asia, North America, South America	[30,60,155,176,177]
Kaiowa virus	Unknown	*Ae*. *aegypti*, *Ae*. *albopictus*, *Cx*. *quinquefasciatus*, *Hg*. *janthinomys*	Asia, Australia, Europe, North America, South America	[32,38,55,60,166,178,179]
Wuhan mosquito virus 6	Orthomyxoviridae	*Ae*. *albopictus*, *Ae*. *cinereus*, *Ae*. spp., *An*. *sinensis*, *Cx*. *australicus*, *Cx*. *globocoxitus*, *Cx*. *modestus*, *Cx*. *pipiens*, *Cx*. *quinquefasciatus*, *Cx*. *tarsalis*, *Cx*. *torrentium*, *Cx*. *tritaeniorhynchus*, *Cx*. spp., *Hg*. *janthinomys*	Asia, Australia, Europe, North America, South America	[30,34,43,55,60,155,163,177]
Aedes flavivirus	Flaviviridae	*Ae*. *aegypti*, *Ae*. *albopictus*, *Ae*. *flavopictus*, *Ae*. *luteocephalus*, *Ae*. *sudanensis*, *Ae*. *tricholabis*, *Ae*. spp., *Cx*. *pipiens*	Asia, Australia, Europe, South America	[49,60,167,168,170,175,178,180–187]
Alphamesonivirus 4	Mesoniviridae	*Ae*. *aegypti*, *Ae*. *notoscriptus*, *Ae*. *procax*, *Ae*. *vigilax*, *Ar*. *subalbatus*, *Cx*. *annulirostris*, *Cx*. *tritaeniorhynchus*	Asia, Australia, South America	[33,42,155,161]
Beaumont virus	Rhabdoviridae	*An*. *annulipes*, *An*. spp., *Cx*. *modestus*, *Cx*. *pipiens*, *Cx*. spp.	Africa, Asia, Australia, Europe	[34,152,188]
Culex flavivirus	Flaviviridae	*Ae*. *aegypti*, *Ae*. *scapularis*, *Ae*. *vexans*, *An*. *sinensis*, *Cx*. *coronator*, *Cx*. *declarator*, *Cx*. *fuscocephala*, *Cx*. *nebulosus*, *Cx*. *nigripalpus*, *Cx*. *pipiens*, *Cx*. *quinquefasciatus*, *Cx*. *restuans*, *Cx*. *tarsalis*, *Cx*. *tritaeniorhynchus*, *Cx*. *univittatus*, *Cx*. *vishnui*, *Cx*. spp.	Africa, Asia, North America, South America	[30,38,40,52,155,159,164,168,169,171,174,175,177,180,182,183,189–204]
Culex iflavi-like virus 4	Iflaviridae	*Ae*. *aegypti*, *Ae*. *albopictus*, *Ae*. *cinereus*, *Cx*. *modestus*, *Cx*. *pipiens*, *Cx*. *quinquefasciatus*, *Cx*. *tarsalis*, *Cx*. spp.	Asia, Europe, North America, South America	[30,34,40,155,166,177,178,205]
Guato virus	Unknown	*Ae*. *albopictus*, *Ae*. *aegypti*, *Hg*. *janthinomys*	Asia, Europe, North America, South America	[38,55,60,166,178]
Hubei chryso-like virus 1	Unknown	*Ae*. *aegypti*, *Ae*. *albopictus*, *Ae*. *cinereus*, *An*. *sinensis*, *Cx*. *australicus*, *Cx*. *globocoxitus*, *Cx*. *modestus*, *Cx*. *pipiens*, *Cx*. *quinquefasciatus*, *Cx*. *tritaeniorhynchus*, *Cx*. spp.	Asia, Australia, Europe, North America	[30,34,50,52,60,163,177,205]
Hubei mosquito virus 2	Unknown	*Ae*. *aegypti*, *Ae*. *albopictus*, *Ae*. *communis*, *Ae*. spp., *An*. *sinensis*, *Ar*. *subalbatus*, *Cx*. *bitaeniorhynchus*, *Cx*. *pipiens*, *Cx*. *quinquefasciatus*, *Cx*. *tritaeniorhynchus*	Asia, Europe, North America, South America	[32,33,37,39,46,50,52,60,166,177,178]
Hubei virga-like virus 2	Unknown	*Ae*. *aegypti*, *Ae*. *albopictus*, *Cx*. *erythrothorax*, *Cx*. *pipiens*, *Cx*. *quinquefasciatus*, *Cx*. *tarsalis*, *Hg*. *janthinomys*	Asia, Europe, North America, South America,	[30,38,55,60,155,177,205]
Humaita-tubiacanga virus	Unknown	*Ae*. *aegypti*, *Hg*. *janthinomys*	Asia, Australia, North America, South America	[32,37,38,55]
Merida virus	Rhabdoviridae	*Ae*. *aegypti*, *Ae*. *albopictus*, *Cx*. *pipiens*, *Cx*. *tarsalis*, *Cx*. *torrentium*, *Cx*. *tritaeniorhynchus*, *Hg*. *janthinomys*	Asia, Europe, North America, South America	[30,43,50,55,60,166]
Wenzhou sobemo-like virus 4	Unknown	*Ae*. *aegypti*, *Ae*. *albopictus*, *Ae*. *communis*, *An*. *sinensis*, *Ar*. *subalbatus*, *Cx*. *quinquefasciatus*, *Cx*. *tritaeniorhynchus*	Asia, Europe, North America, South America	[30,32,33,37,46,50,60,166,178]
Whidbey virus	Orthomyxoviridae	*Ae*. *aegypti*, *Ae*. *albopictus*, *Ae*. *cantans*, *Ae*. *cinereus*, *Cx*. *modestus*, *Cx*. *pipiens*, *Cx*. spp., *Ma*. *wilsoni*	Asia, Australia, Europe, South America	[32,34,46,166,178,206]
Wuhan mosquito virus 8	Chuviridae	*Ae*. *aegypti*, *Cx*. *pipiens*, *Cx*. *quinquefasciatus*, *Cx*. *tritaeniorhynchus*	Asia, Europe, North America, South America	[33,37,38,50,155,177]
Wuhan mosquito virus 9	Rhabdoviridae	*Ae*. *aegypti*, *An*. *sinensis*, *An*. spp., *Cx*. *pipiens*, *Cx*. *quinquefasciatus*, *Cx*. *tritaeniorhynchus*	Africa, Asia, Australia, South America	[32,33,50,152,155,177]

While the increase in the number of metagenomic studies has allowed for the description of more viral species, it has also shown that our understanding of viral ecology is incomplete and heavily biased towards pathogenic viruses that directly impact humans [25,28,29]. Even in relatively well-described mosquito systems (e.g., *Ae*. *aegypti*), much is unknown about most of the viruses identified. A few studies have looked at transmission pathways of known ISVs [62–65], and some of these have begun examining environmental associations [56] and fitness costs [62,66]. However, additional research will be required to determine transmission pathways, interactions with the host and coinfecting microbes, and the effect of the host’s environment for novel viruses. For many other common ISVs, transmission pathways have been theorized based on the ecology of related viruses or observations of persistence in long-term lab colonies, but these pathways have not been experimentally confirmed. In addition, the fitness costs of most ISVs have yet to be described, and it is unknown if they are pathogenic to their mosquito host. Fitness costs associated with infection are expected to significantly vary with environmental context and could be a driver of virus and host abundance across a landscape [67]. In addition, interactions among viruses have only begun to be explored and only in the context of interactions with pathogenic viruses (e.g., dengue) [48,68,69].

Sequencing the viromes of individual mosquitoes [30,38] has allowed researchers to examine how ISVs vary among individual mosquitoes and has opened up the possibility of exploring the cause, extent, and impact of variation in virus composition. Importantly, researchers can now look not only at viral diversity but also at the genetic variation in individual viruses within and among hosts that results from eco-evolutionary forces acting on both the virus and mosquito host [70]. This information is important for understanding the risk of ISVs evolving pathogenicity in novel hosts [71–73]. While host-switching is vanishingly rare, genetic variation within ISVs allows researchers to target specific viruses with phylogeographic methods to quantify their transmission and dispersal [70,74]. Given that species-specific viruses, or at least viral lineages, are likely to exist, this could provide methods to indirectly estimate mosquito dispersal or population dynamics. In particular, the genomes of maternally inherited ISVs could provide alternatives to mitochondrial genes for mosquito population genetic studies and could be used within a phylodynamic framework to estimate mosquito population dynamics.

## Epidemiological modeling of ISV ecology

Due, in part, to recent high-profile pandemics (e.g., Influenza, Ebola, and COVID-19), there has been increased interest in the development of quantitative methods for analyzing the spatiotemporal dynamics of viral outbreaks [75,76]. These methods can be broadly divided into spatial and genomic epidemiology. While developed for examining the dynamics associated with pathogens of medical and veterinary relevance, they are equally applicable to ISVs provided method-specific conditions are met. The field of spatial epidemiology applies linear models, scan statistics, and geographic profiling to disease occurrence data (e.g., counts of infected individuals be location/region) to determine the possible environmental drivers of disease seasonally and across geographic regions. Genomic epidemiology, on the other hand, leverages sequencing data from individuals to reconstruct viral phylogenic histories, where each inferred ancestral node has a set of characteristics (e.g., time and location). Assuming the population is measurably evolving, a molecular clock model combined with a sampling location and time can be used to estimate (1) population growth rate [77–79]; (2) dispersal rate across a landscape [80]; or (3) movement between populations [70,74]. As described below, these epidemiological models can be easily adapted to viruses present in the mosquito virome (**Fig 1**).

### Spatial epidemiology

Numerous methods exist for quantifying the role of environmental variation and host movement on the spatial and temporal patterns of disease outbreaks. Techniques such as scan statistics and geographic profiling have been developed to identify likely sources of infection or infection-spreading agents [81–83]. These techniques can be used to estimate host dispersal and identify potential sources of uncommon environmentally acquired viruses (either from host blood meals, plant sources, or other areas (e.g., larval habitat)), as they would be expected to cluster around the source of infection. Scan statistics compare the frequency of incidence of an event within a geographic area around each event to a null expectation of the frequency predicted from a random point process (e.g., a Poisson process) [81,84]. Deviations from the null expectation identify spatiotemporal clustering. These clusters can then be linked to known spatial features (e.g., water sources) or events (e.g., flooding). Similarly, geographic profiling attempts to locate the source of an event, such as an infection, by assuming that outbreaks are rare events that originate from very few sources, with a dispersal kernel for the spreading agent (e.g., host movement) and a likelihood for the spatial location of the source simultaneously estimated [82]. It is then possible to determine the most likely number of sources and their locations by calculating maximum likelihoods for varying numbers of sources. This approach benefits from a Bayesian framework, allowing for the incorporation of prior knowledge about potential sources of infection. Geographic profiling has been used to locate larval habitat of malaria vectors from human malaria cases [82].

### Genomic epidemiology

Models deployed in genomic epidemiology, such as phylogeography [85,86] and phylodynamics [70,87,88], use the phylogeny of a clade, often an individual species or virus, to estimate epidemiologically important parameters. This occurs in 2 steps: (1) estimating a phylogeny based on genetic variation between individuals using any number of methods (e.g., maximum-likelihood or Bayesian estimation); and (2) estimating parameters of interest from the phylogeny using simulation-based methods (e.g., Approximate Bayesian Computation) [70]. Since genomic epidemiology is regularly done with RNA viruses, modeling of ISVs would be relatively straightforward given an appropriate virus. Many of the typically estimated parameters in these models may not be of direct interest (e.g., ISV introduction rate), but they can be informative if they are indicative of an aspect of mosquito ecology (e.g., mosquito immigration). While both methods use a viral phylogeny as the basis for inference, their aims, methods, and estimated parameters differ.

Phylogeography aims to estimate the dispersal rate of an organism across space based on the distance between individuals and the time since their most recent common ancestor by simultaneously simulating models of spread and reconstructing the evolutionary history based on genomic data [86]. If the target of phylogeographic models cannot actively disperse, like pathogens, inference can be made about the species responsible for their spread, as has been done with mosquito-borne diseases previously [85,89–94]. Phylogeographic models of dengue virus have been used to implicate mosquito dispersal [91] and human population density [90] as the primary drivers of clustering within a city and air travel as the primary driver of intercontinental spread [92]. Additionally, phylogeographic analysis of West Nile virus has shown nonhomogenous spread across North America [93,94]. Despite these successes, phylogeographic studies have been limited by their reliance on sequences from pathogenic viruses collected from non-mosquito hosts. The identification of suitable target ISVs can expand the usefulness of phylogeographic methods by allowing them to be applied outside of epidemic settings, removing complications associated with a multiple-host transmission pathway, and increasing sample sizes. Ideal target viruses for these methods would have sufficiently high evolutionary rates [70,95], be species specific, and have sufficient prevalence to allow for collection.

Phylodynamics is the study of how epidemiological, immunological, and evolutionary processes act on phylogenies [70]. This can either be done by estimating changes in the effective population size via coalescence models (e.g., skyline models) [77–79] or estimating relevant parameters using mechanistic viral transmission models (e.g., an SIR model) [70,74]. Phylodynamic methods have previously been used to estimate fluctuations in viral effective population sizes [96–99], *R*_*0*_ [100–107], pathogen introduction rates [103,107], and pathogen transmission between groups and locations [100,108–110]. Similar to phylogeographic studies, phylodynamic studies have been largely limited to pathogenic RNA viruses, where sequencing of isolates have become commonplace, especially during ongoing epidemics (e.g., SARS-CoV-2). These frameworks can also be easily adapted to target ISVs. For core ISVs, or others that maintain a relatively constant prevalence, changes in the host population size could be inferred from changes in the viral population size. Ideally, target viruses would have the same attributes as for phylogeographic models, with the addition of a known transmission pathway if a mechanistic model is used. However, previous work has shown that mechanistic methods can determine the relative importance of different modes of transmission [111], opening the possibility of using phylodynamic models to infer unknown transmission pathways. Of particular interest would be maternally inherited ISVs, where viral dynamics would be closely tied to host dynamics due to transmission only occurring when infected hosts are born.

## Epidemiological modeling of ISVs to understand mosquito-borne disease ecology

**Fig 2:**
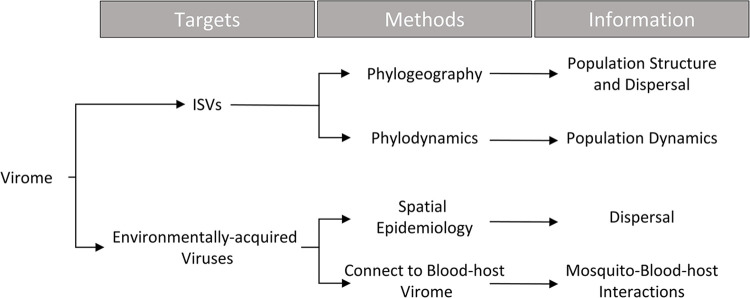
Viruses have the potential to elucidate several aspects of mosquito and mosquito-borne disease ecology. By combining viral phylogenies and incidence data with appropriate methods, it will be possible to describe mosquito population dynamics and structure, dispersal rates, and mosquito–blood–host interactions in the field.

While previous epidemiological modeling has been pathogen focused, several methods have been developed to describe the host population based on its effect on pathogen incidence and phylogenies. While of little interest in systems where the host population can be directly observed (e.g., humans), this opens the possibility of estimating population structure and dynamics when the host population cannot be directly observed (e.g., mosquitoes). ISVs are abundant within mosquito species and when studied within an epidemiological modeling framework will allow us to estimate several aspects of mosquito-borne disease ecology. **Fig 2** provides a diagram tying elements of the virome to various aspects of mosquito-borne disease ecology through the appropriate modeling framework.

### Population dynamics

Estimating changes in mosquito abundance on epidemiologically important time scales (e.g., weeks to months), and relating those changes to disease incidence has proven difficult. Virome diversity and viral genetic diversity are intrinsically linked to these dynamics and measurably change on these timescales [70], providing a possible avenue for estimating dynamics at fine timescales. Previous methods for estimating mosquito population sizes have relied on mosquito trapping rates or the frequency of mosquito landing on “bait” hosts, producing estimates of relative mosquito abundance between sites. Genetics-based techniques have also been used to estimate the effective mosquito population size. However, these techniques can only estimate the (relative or effective) population size at a particular time point and are less accurate if the population is growing or shrinking. Phylodynamic models of ISVs provide a method of estimating these dynamics [70]. Mechanistic models of viral transmission can be incorporated into phylodynamic models to estimate relevant parameters (e.g., transmission rates, effective population size, and *R*_*0*_) by simulating viral phylogenies and estimating marginal likelihoods of the observed phylogeny. For maternally inherited ISVs, where new infections occur when a host is born and persist until host death, viral infection rates and population sizes are roughly proportional to the host birth rate and population size. Similarly, for an endemic ISV with a relatively constant prevalence in the population, changes in the effective population size of the virus would be indicative of changes in the host population. In addition, recently developed methods incorporating other sources of data (e.g., incidence data) into these methods result in improve estimates of effective population sizes and growth rates [112], allowing for the inclusion of trapping or host landing count data into the model.

### Mosquito movement and population structure

Mosquito dispersal and population structure is strongly affected by landcover, with roads [113,114] and rivers [115] serving as both barriers to and pathways for dispersal. The presence of barriers can result in limited gene flow between proximate populations and can result in fine scale population structure (e.g., <1 km). This is especially true for weak active dispersers like *Ae*. *aegypti*, which rarely disperses more than 200 m in its lifetime [20–24]. Mosquito population structure is further complicated by passive dispersal of mosquitoes by humans inadvertently transporting adults (e.g., by car/airplane) or eggs (e.g., tires/bamboo plants) [116]. The resulting population structure, as well as the drivers of this structure, have major implications for mosquito-borne disease spread and vector control. Connectivity between populations determines the area over which mosquitoes can spread disease [6–8], and microgeographic adaptation may occur in genetically isolated populations under unique selection pressures [117,118] resulting in variation in disease risk across landscapes [1]. This variation, in turn, can challenge our ability to accurately predict geographic disease risk and the impacts of climate change [4]. Further, population structure affects the success of mosquito reduction programs [15,119], the spread of important alleles (e.g., knock-down resistance) [120–122], and reinvasion risk [11–13]. Finally, it can be difficult to quantify the relative importance of environmental barriers compared to geographic distance in isolating populations [80,123].

Interest in estimating population structure and determining barriers to gene flow in mosquito populations has increased following high-profile releases of *Ae*. *aegypti* transinfected with *Wolbachia* [14,16,124]. Yet dispersal, population structure, and the effect of environmental variation on epidemiologically relevant scales remain poorly understood. Traditional methods of quantifying movement of individuals (e.g., mark-release-recapture) have substantial drawbacks when working with mosquito species. Mark-release-recapture (MRR) methods (e.g., fluorescent dye) are logistically straightforward and commonly used but have low recapture rates resulting in low confidence in dispersal estimates obtained [20,24,125,126]. This has led to the development of additional methods for evaluating dispersal and local population structure, including the use of stable isotope markers [22,127], microcrystals [128], landscape genomics [129], and close-kin mark recapture (CKMR) [10,23,130]. However, each of these have major limitations when estimating population structure. The use of stable isotope markers, which introduces either ^13^C or ^15^N isotopes to larval habitat to mark mosquitoes, has been limited by a lack of available markers and low capture rates. Landscape genomics, which estimates relatedness between populations based on genetic variation, has difficulty resolving the local population structure that is important for epidemiological processes [113,114,131,132] due to slow substitution rates and gene flow [113,133]. CKMR, which estimates mosquito movement at fine spatial scales using the location and timing of captured close-kin (e.g., third degree or closer relatives), requires the capture of several close-kin pairs. The intensive trapping required to capture pairs [10,23,130], however, makes scaling CKMR methods to regional or intermediate spatial scales (e.g., cities) essentially impossible. In addition, CKMR is likely to miss long-distance dispersal events due to requiring the capture of close kin of the disperser in both its source and terminal location.

**Fig 3:**
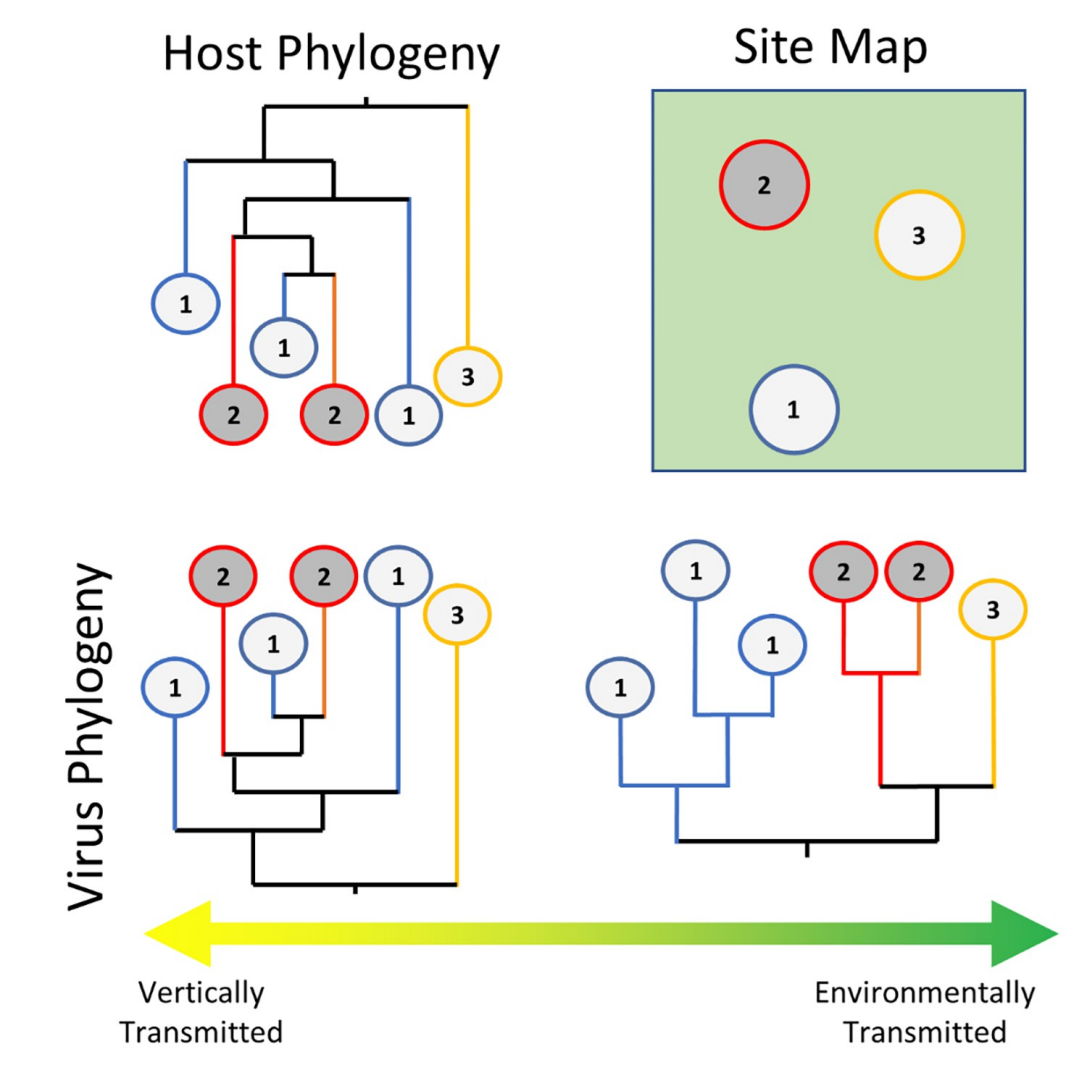
Viral phylogenies are reflective of transmission pathways. Theoretical phylogenies of a host sampled from 3 locations and 2 viruses are given. Phylogenies of exclusively maternally transmitted viruses are expected to reflect the host’s genealogy and the resultant phylogeny, while those that are solely acquired from the environment are expected to group according to their sampling location. From this, it may be possible to infer dominant transmission pathways based on viral phylogeny. If the transmission mode is known, deviation from the expected phylogeny can provide evidence of other dynamics including host movement between sites.

Leveraging information from the mosquito virome, particularly species-specific ISVs, will allow studies to apply a uniform framework to simultaneously examine mosquito dispersal across multiple spatial scales (e.g., between backyards, cities, regions). Species-specific ISVs are expected to have the same population structure as their hosts (**Fig 3**). If these viruses also have sufficient genetic variation and high mutation rates (i.e., higher than host mitochondrial genes), applying phylogeographic methods of ISV genetic variation should be capable of estimating mosquito dispersal rates at both fine and coarse spatial scales. Similarly, spatial clustering in viral phylogenies are known to be indicative of host population structure due to isolation of viral lineages [70]. Given the higher mutation rates in ISVs, phylogeny-based methods using sequence variation among ISVs would allow for finer scale resolution of population structure (without the need for capturing close-kin) with the appearance of new, distantly related viral lineages indicative of host immigration [70,94]. Increased between-site beta diversity in the virome would also be indicative of host population structure, as limited movement between sites restricts environmentally acquired viruses (e.g., from sugar-feeding). Preliminary work examining the phylogeography of ISVs has been able to discern movement between continents and species but showed differing results between results from different ISVs [134]. However, these results were based on relatively small sample sizes (*n =* 18 to 46) taken from multiple mosquito genera.

Targeting ISVs as opposed to genetic information in the host’s genome will allow for the use of modern molecular epidemiological methods to directly estimate mosquito movement rates in real time, which are needed to parameterize disease transmission models and optimize intervention strategies. These methods cannot be used with host genomic information due to high rates of recombination [135,136]. Similar molecular epidemiological methods based on viral phylogenies have previously been used for inference at various spatial scales to estimate the dispersal rate of rabies [137] and WNV [94] across a landscape and the rate of viral introductions at a site [103,107].

### Current limitations

While ISVs have the potential to expand our knowledge of mosquito-borne disease ecology, this potential is currently limited by a lack of known target ISVs. This is further complicated by our limited understanding of how complex interactions with other endosymbionts (both viral and bacterial) affect ISV eco-evolutionary dynamics and, hence, inference regarding host populations. Several studies examining phylogenies of ISVs demonstrated to be vertically maintained in the laboratory have also shown evidence of horizontal transmission in the field [138–140], highlighting the challenge in choosing appropriate target ISVs for a given study. Additionally, several studies have shown interactions of ISVs with other viruses [66,141] and the bacterial endosymbiont *Wolbachia* [139,142–144]. This compounds the difficulty of identifying appropriate target ISVs, especially when viral and bacterial communities vary among populations. Further, with ongoing releases of *Wolbachia*-transinfected mosquitoes, it is possible that ISVs that serve as appropriate targets prerelease may be less suitable following the release if the target ISVs interact with *Wolbachia*. If appropriate target virus cannot be identified, other methods (e.g., geographic profiling) may prove more suitable than phylogeographic methods for estimating mosquito dispersal at local scales. As studies continue to empirically test transmission pathways and determine phylogenies with increased accuracy, appropriate initial target ISVs should become apparent. For this reason, it is important to identify viruses (or viral strains) that are species specific, are sufficiently prevalent, and have phylogenies that correlate well with host phylogenies within the study region. Table 1 provides a list of widespread ISVs that could serve as an initial list for evaluation as targets. While it may be possible to identify candidate ISVs that are broadly appropriate, it is likely that the choice of virus will need to be site, system, and question specific.

### Sample sizes and power calculations

Incorporating the virome into mosquito-borne disease ecology studies will require careful consideration of the sample sizes required for studies to be appropriately powered. Sample sizes will be constrained by the high costs of mNGS. While the cost of sequencing has fallen since the development of mNGS and is likely to continue to decrease, sequencing 100 individuals at 10 million reads per individual [38] still costs nearly $8,000 in 2022 [145]. If target viruses are known before sequencing, multiplexed targeted amplicon sequencing (TAS) can be used instead. This has the benefit of greatly reducing the cost per sample [146,147] and can be integrated into ongoing arbovirus surveillance. However, TAS will be poor at quantifying virome diversity. In addition, determining appropriate sample sizes requires preliminary estimates of key parameters that should be empirically informed. Power estimates for studies using virome diversity would require a preliminary estimate of variation in virome composition. Similarly, power estimates using spatial or molecular epidemiological models would require estimates of viral prevalence and power estimates for epidemiological models would also require estimates of viral molecular clocks. Once reasonable estimates are available, simulation-based power studies [129,148], similar to previous landscape genomic studies [149–151], can provide a guide to best practices in determining sampling locations, timing, and sizes minimizing the amount of sampling, and sequencing, required.

### Conclusions

The mosquito virome has the potential to revolutionize our understanding of mosquito-borne disease ecology. Through epidemiological modeling of ISVs, it is possible to begin quantifying many previously cryptic aspects of mosquito-borne disease ecology. In particular, ISVs provide a means for employing phylogeographic and phylodynamic models to estimate host population movement, both between and within sites. These rates are key to efficient mosquito control programs, especially as the use of *Wolbachia* as a biocontrol and other genetic interventions become more common.
